# Resistance training improves sleep quality, redox balance and inflammatory profile in maintenance hemodialysis patients: a randomized controlled trial

**DOI:** 10.1038/s41598-020-68602-1

**Published:** 2020-07-16

**Authors:** Hugo Luca Corrêa, Sting Ray Gouveia Moura, Rodrigo Vanerson Passos Neves, Carmen Tzanno-Martins, Michel Kendy Souza, Anderson Sola Haro, Fernando Costa, José Adeirton Bezerra Silva, Whitley Stone, Fernando Sousa Honorato, Lysleine Alves Deus, Jonato Prestes, Herbert Gustavo Simões, Elaine Cristina Vieira, Gislane Ferreira de Melo, Milton Rocha Moraes, Thiago Santos Rosa

**Affiliations:** 1 Graduate Program of Physical Education, Catholic University of Brasilia (USB), EPTC, QS07, LT1 s/n. Bloco G Sala 117, Águas Claras, Taguatinga, Brasília, DF 71966-700 Brazil; 2 HDC RenalClass, São Paulo, SP Brazil; 3 Department of Nephrology, Federal University of São Paulo, São Paulo, Brazil; 4 School of Kinesiology, Recreation, and Sport, Western Kentucky University, Bowling Green, KY USA

**Keywords:** Nephrology, Kidney, Kidney diseases, Physiology, Kidney

## Abstract

Patients in maintenance hemodialisys (HD) present sleep disorders, increased inflammation, unbalanced redox profiles, and elevated biomarkers representing endothelial dysfunction. Resistance training (RT) has shown to mitigate the loss of muscle mass, strength, improve inflammatory profiles, and endothelial function while decreasing oxidative stress for those in HD. However, the relation between those factors and sleep quality are inadequately described. The aim of this study was to verify the effects of 3 months of RT on sleep quality, redox balance, nitric oxide (NO) bioavailability, inflammation profile, and asymmetric dimethylarginine (ADMA) in patients undergoing HD. Our primary goal was to describe the role of RT on sleep quality. Our secondary goal was to evaluate the effect of RT on NO, metabolism markers, and inflammatory and redox profiles as potential mechanisms to explain RT—induced sleep quality changes. Fifty-five men undergoing maintenance hemodialysis were randomized into either a control (CTL, n = 25) and RT group (RTG; n = 30). Participants in the RT group demonstrated an improvement in sleep pattern, redox, inflammatory profiles, and biomarkers of endothelial function (NO_2_^−^ and ADMA). This group also increased muscle strength (total workload in RT exercises of upper and lower limbs). These findings support that RT may improve the clinical status of HD patients by improving their sleep quality, oxidative and inflammatory parameters.

## Introduction

Sleep disorders have shown to negatively influence inflammation, oxidative stress, and nitric oxide (NO). Because of this, there has been a growing health concern for patients with sleep disturbance in maintenance hemodialysis (HD) due to chronic kidney disease (CKD). This multisystemic condition is considered a public health problem as the pathologies associated with CKD decrease kidney function^1^. Specifically, the fifth, and final, stage of CKD requires invasive treatment because approximately 90% of the nephrons are not functional^1,2^. To compound the issue, the progression of CKD is associated with worsening sleep disorders and other comorbidities associated with the loss of renal function^3^. It could then be postulated that improving sleep quality would contribute positively to life expectancy and quality of life in a population with CKD.

Sleep is a natural phenomenon that is characterized by a state of altered consciousness, reduced interactions with the environment, and attenuated muscle activity. Sufficient sleep is essential for health as it facilitates several physiologic processes, such as memory consolidation, clearance of brain metabolites, and recovery of nervous, immune, skeletal, and muscular systems. Risk for metabolic diseases^4^ and neuronal protein synthesis impacting learning and memory^5^ are examples of systems and processes that are are negatively impacted by poor sleep. Kidney function and the excretion of hormones (involved in cell communication) seems to be modulated by the sleep quality^4,6^. In this regard, sleep disorders can be a risk factor for CKD and potentially contribute to the progression of the early stages of CKD^3^. Moreover, NO has been demonstrated an important role in sleep modulation, while the application of the inhibitor of the enzymes responsible for NO production (L-NAME) reduces sleep quality^7^.

To test this theory, Solak et al.^8^ compared the effects of sildenafil (NO donor) on depression and sleep quality in patients undergoing HD. The authors verified that both medications improved sleep quality and decreased depression symptoms in this population, highlighting a clue for the role of NO on sleep quality. Hirotsu et al.^9^ evaluated chronic sildenafil treatment in an animal model of CKD submitted to sleep deprivation. Despite sleep withdrawal, the authors found that the medication increased NO bioavailability (a vasodilator), and induced reno-protection^10^. Taken together, these evidences point to a possible influence of NO on renal integrity and also, on the modulation of encephalic centers of sleep.

Resistance training (RT) has been shown to evoke NO release, reduce asymmetric dimethylarginine (ADMA; endogenous inhibitor of nitric oxide synthases), and improve redox and inflammatory profiles^10–15^. If nitric oxide is a relevant pathway to improve sleep and renal function through positive inflammatory responses, chronic resistance training (RT) may be a potent non-pharmacological tool to enhance the quality of sleep in HD patients. These physiological modifications could mediate crosstalk between physical activity and sleep quality. However, there is little evidence to support the efficacy of exercise in sleep quality for HD patients^16^. Other exercise modalities (aerobic, RT and combined training) have shown to exert benefits in sleep quality in end-stage renal disease patients^11^. The specific mechanisms explaining the improvement of sleep pattern in this population remain to be determined.

Moreover, Gopaluni et al.^17^, concluded in their review that additional studies are required to investigate the effect of exercise programs with intradialytic intervention in the improvement of sleep disorders. In a recent review, Bohm et al.^18^ also demonstrated few studies with intradialytic exercise and sleep quality. Also, part of these studies had as primary outcome glycemic and/or lipid homeostasis. Therefore, remains unknow whether the inflammatory variables or NO bioavailability can explain the primary outcome.

The presented gap in the literature prompted the current study, aimed at evaluating the effects of RT for 3 months on sleep quality, redox balance, inflammation, NO, and ADMA in HD patients. We hypothesized that intradialytic RT would improve sleep quality. Moreover, we hoped to verify the involvement NO bioavailability, decrease of ADMA concentration, and the regulation of redox balance and inflammatory profile as mechanisms for change in sleep quality in the studied population. Our primary purpose was to evaluate the role of RT on sleep quality, with a secondary aim of investigating the effect of RT on NO, metabolism markers, and inflammatory and redox profiles as potential mechanisms to explain RT—induced sleep quality changes.

## Results

There was no difference between groups for baseline age, body weight, height, body mass index (BMI), hemodialysis (HD) time, HD period, and smoking (*p* > 0.05). See Table 1

**Table 1:** Baseline characteristics of patients.

Variables	CTL (*n* = 25)	RT (*n* = 30)	*p* value
Age (years)	65.7 ± 3.8	66.0 ± 4.0	0.8164
Body weight (kg)	74 ± 7	75 ± 7	0.7755
Heigth (m)	1.72 ± 0.07	1.74 ± 0.07	0.2498
BMI (kg/m^2^)	25.2 ± 2.1	24.7 ± 1.7	0.3358
HD time (months)	59.8 ± 7.7	60.7 ± 8.0	0.6481
HD period			
Morning, *n*	8	11	0.7171
Afternoon, *n*	7	9	0.8708
Night, *n*	10	10	0.6088
Smokers, *n*	7	9	0.8708
Sex, % of men	100	100	-

Data demonstrated in mean ± SD.

*CTL* control group, *RT* resistance training group, *BMI* body mass index, *HD* hemodialysis. Chi-square test was used to analyze the baseline characteristics of individuals.

**Figure 1:**
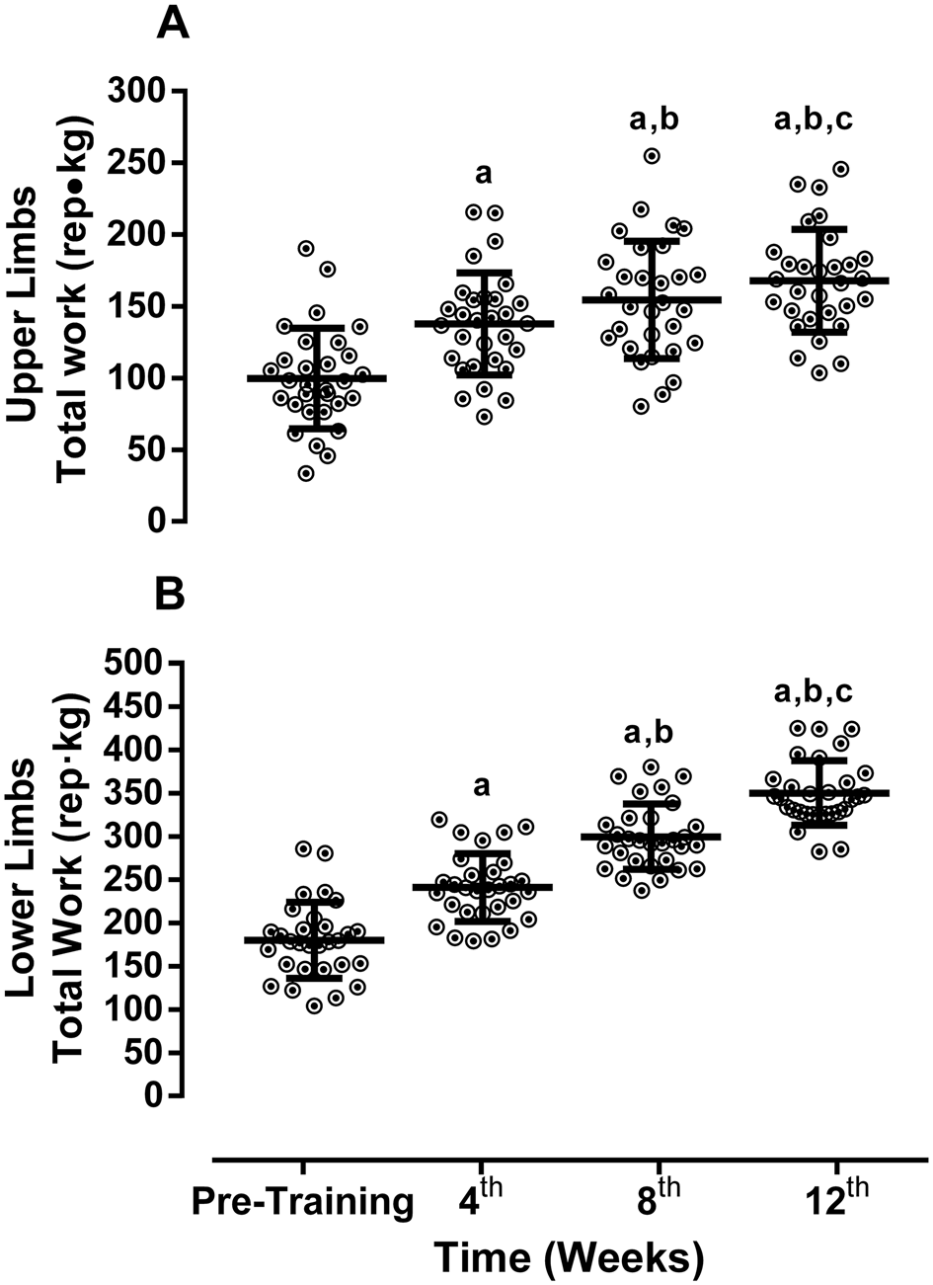
Total workload. Data demonstrated by mean ± SD. ANOVA-one way of repeated measures followed the Tuckey’s post-hoc test was utilizated to verify differences between weeks. ^a^*p* < 0.05 versus pre-training; ^b^*p* < 0.05 versus 4th week; ^c^*p* < 0.05 versus 8th week.

There were no group × time interactions for iron, albumin, urea pre-dialysis and post-dialysis, potassium and calcium (*p* > 0.05). There was a time effect for hemoglobin, wherein the CTL group saw a marked decrease from pre- to post-intervention (*p* = 0.0054). RTG decreased ferritin across time (*p* = 0.0071) and there was a time effect for phosphate (reduction in both groups; *p* < 0.01). All other biochemical variables were statistically not different between the groups or after training. See Table 2. In regard to training volume, the RTG complied with the progressive overload exercise prescription by marked increases in upper and lower limbs total load across the intervention (*p* < 0.0001). See Fig. 1.

**Table 2:** Pre- and post-training biochemical analysis.

Variables	CTL (*n* = 25)	RT (*n* = 30)	*p* value-interaction
Hemoglobin, g/Dl—pre-intervention	11.2 ± 0.9	10.9 ± 0.6	**0.0054**^a^
Hemoglobin, g/dL—post-intervention	**10.6 ± 0.5**^**b**^	10.7 ± 0.9	
Ferritin, ng/mL—pre-intervention	242.7 ± 102.3	230.4 ± 78.5	**0.0071**^a^
Ferritin, ng/Ml—post-intervention	245.8 ± 110.1	**174.0 ± 58.1**^**c**^	
Albumin, g/dL—pre-intervention	4.1 ± 0.5	4.1 ± 0.7	0.7846
Albumin, g/dL—post-intervention	4.0 ± 0.6	4.0 ± 0.6	
Urea pre-HD, mg/dL—Pre-intervention	106.2 ± 26.9	100.9 ± 19.7	0.3978
Urea pre-HD, mg/dL—post-intervention	101.2 ± 26.3	101.7 ± 24.5	
Urea post-HD, mg/dL—pre-intervention	53.1 ± 11.3	50.6 ± 10.3	0.9684
Urea post-HD, mg/dL—post-intervention	55.0 ± 11.8	52.4 ± 10.1	
Potassium, mEq/L—Pre-intervention	5.2 ± 0.6	5.4 ± 1.0	0.3716
Potassium, mEq/L—post-intervention	5.4 ± 0.6	5.5 ± 0.6	
Phosphate, mg/dL—pre-intervention	5.7 ± 0.6	5.5 ± 0.8	**< 0.01**^a^
Phosphate, mg/dL—post-intervention	**5.0 ± 0.8**^**b**^	**5.0 ± 0.9**^**b**^	

Data demonstrated in mean ± SD. Two-way ANOVA including within and between groups analysis followed by the Tukey’s post-hoc test was adopted to compare the biochemical analysis. Values in bold highlight the significance established by *p* < 0.05

*CTL* control group, *RT* resistance training group, *pre-HD* urea collected before hemodialysis session, *post-HD* urea collected after hemodialysis session.

^a^Group versus time interaction.

^b^Versus Pre-intervention (time effect).

^c^Versus CTL (group effect).

**Figure 2:**
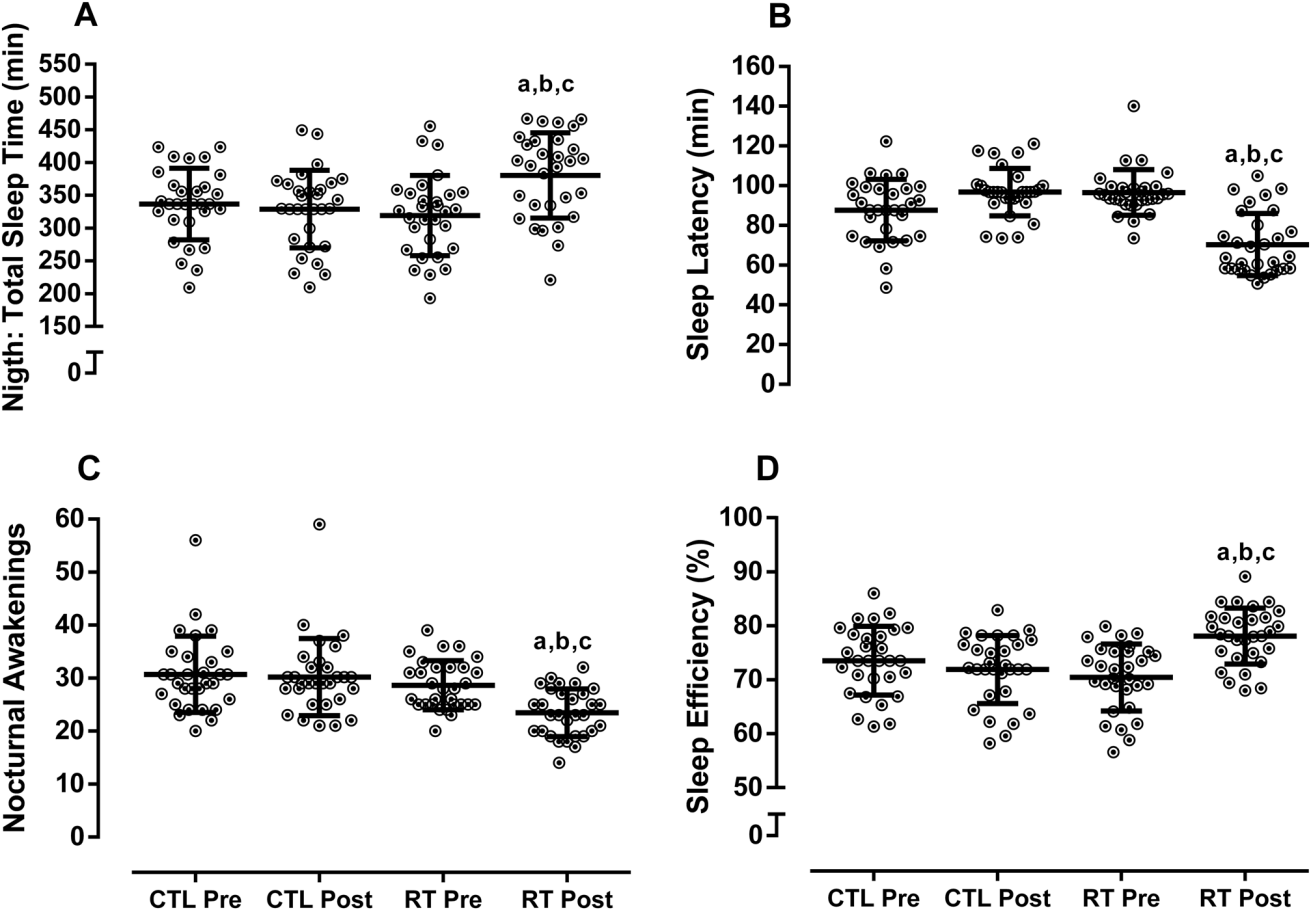
Sleep patterns. Data demonstrated by mean ± SD. ANOVA-two way followed the Tuckey’s post-hoc test was utilizated to verify differences between group and time. *CTL* control, *RT* resistance training. ^a^*p* < 0.05 versus CTL-pre; ^b^*p* < 0.05 versus CTL-post; ^c^*p* < 0.05 versus RT-pre.

There was group × time interaction accelerometer data: total sleep time and sleep efficiency improved in the RTG as compared with pre-training and CTL group (*p* < 0.05). Post hoc analyses indicated that the significant interaction for time and group was present due to decreased nocturnal awakenings and sleep latency for RTG when comparing with pre-training RTG and CTL (*p* < 0.0001). See Fig. 2.

**Figure 3:**
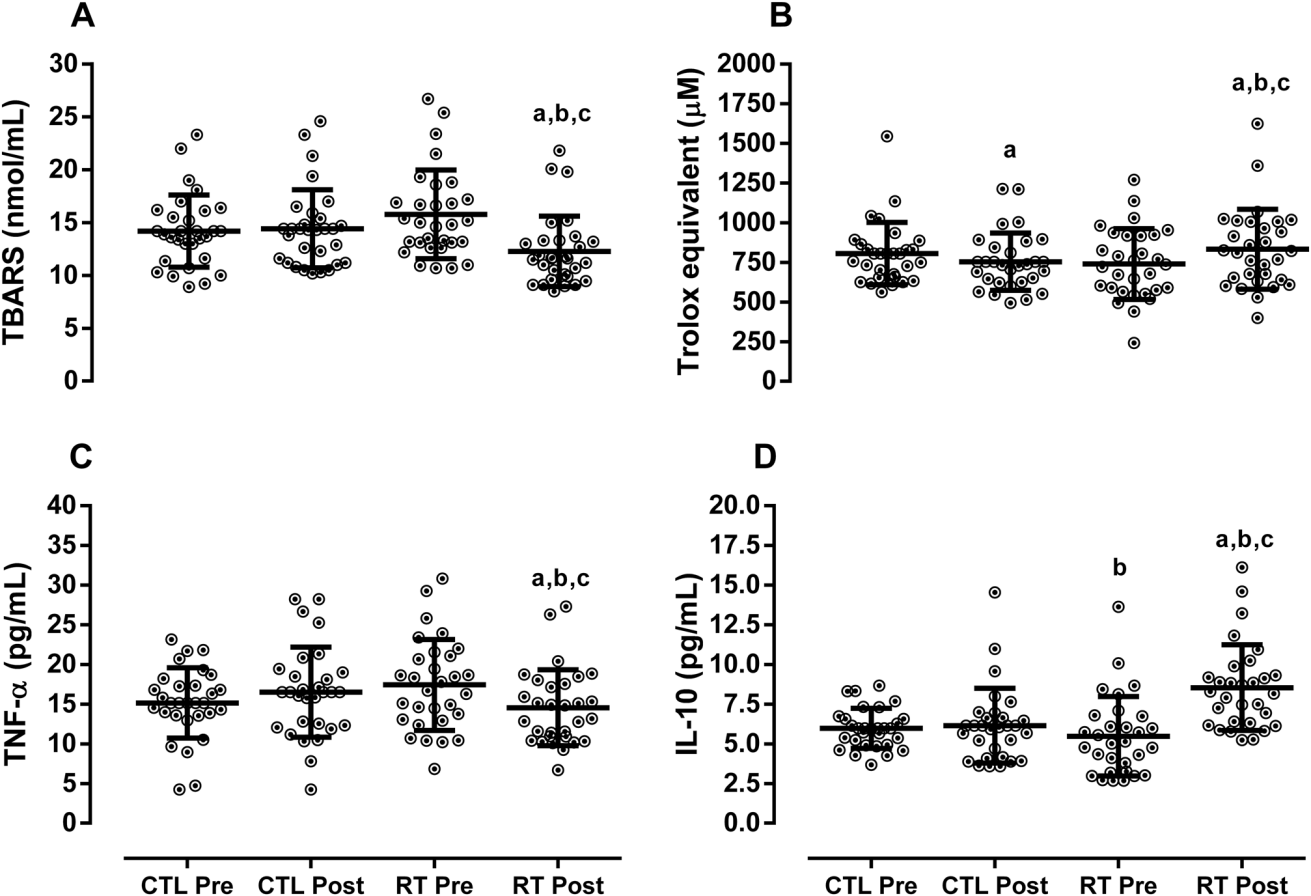
Redox and inflammatory profiles. Data demonstrated by mean ± SD. ANOVA-two way followed the Tuckey’s post-hoc test was utilizated to verify differences between group and time. *CTL* control, *RT* resistance training, *TBARS* tiubarbituric acid reactive substances, *TNF-α* tumor necrosis factor alpha, *IL-10* interleukin 10. ^a^*p* < 0.05 versus CTL-pre; ^b^*p* < 0.05 versus CTL-post; ^c^*p* < 0.05 versus RT-pre.

There was a significant group × time interaction for redox and inflammatory profiles. TBARS and TNF-α decreased, while total antioxidant capacity (Trolox equivalent) and IL-10 increased in the RTG at post-training as compared with pre-training and CTL group (*p* < 0.0001). The CTL participants also experienced a decrease in Trolox (*p* < 0.0001). See Fig. 3.

**Figure 4:**
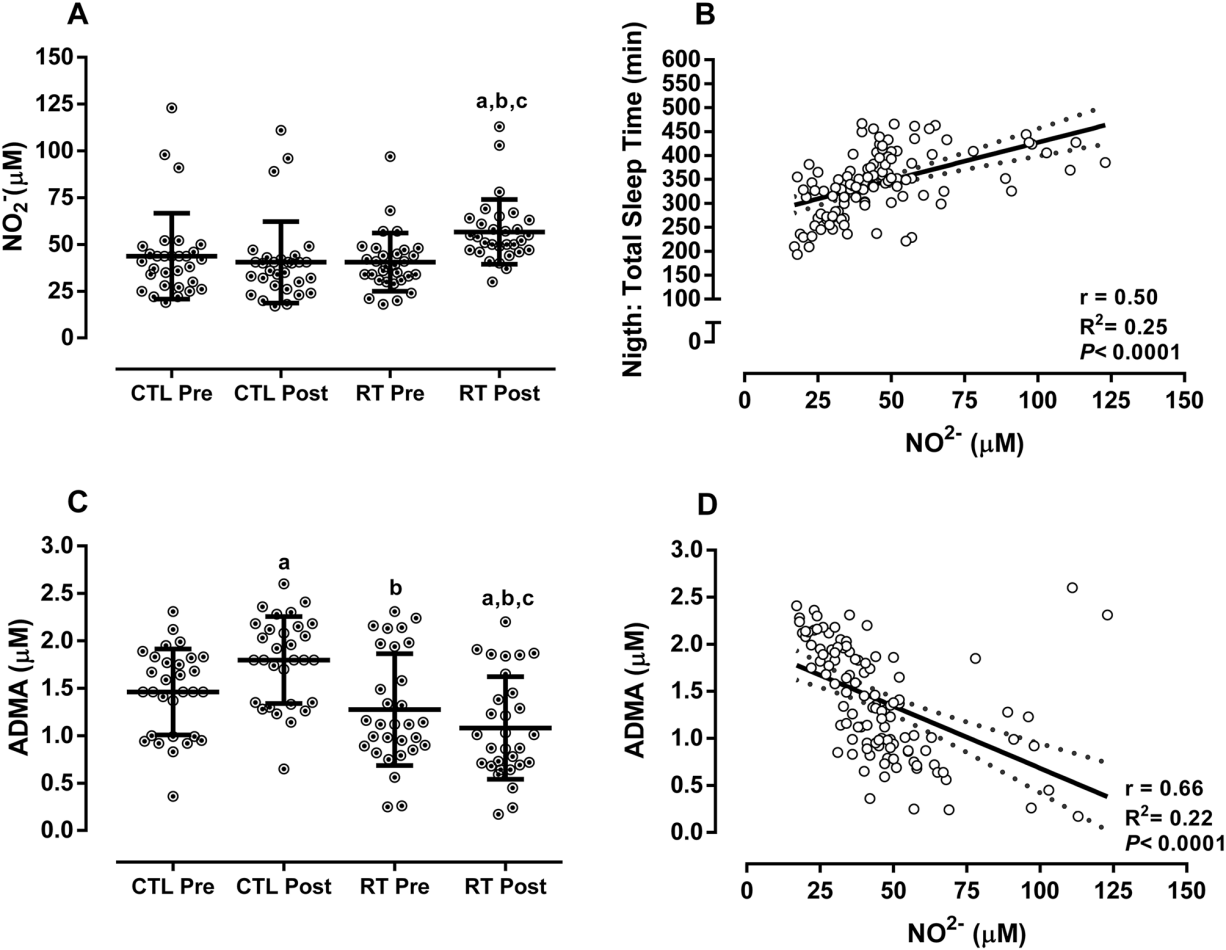
Biomarkers of endothelial function and associations. Data demonstrated by mean ± SD. ANOVA-two way followed the Tuckey’s post-hoc test was utilizated to verify differences between group and time. Pearson correlation coefficients to verify association between total sleep time with NO and ADMA and NO. *CTL* control, *RT* resistance training, *NO*_*2*_^*−*^ nitrite, *ADMA* asymmetric dimethylarginine. ^a^*p* < 0.05 versus CTL-pre; ^b^*p* < 0.05 versus CTL-post; ^c^*p* < 0.05 versus RT-pre.

**Figure 5:**
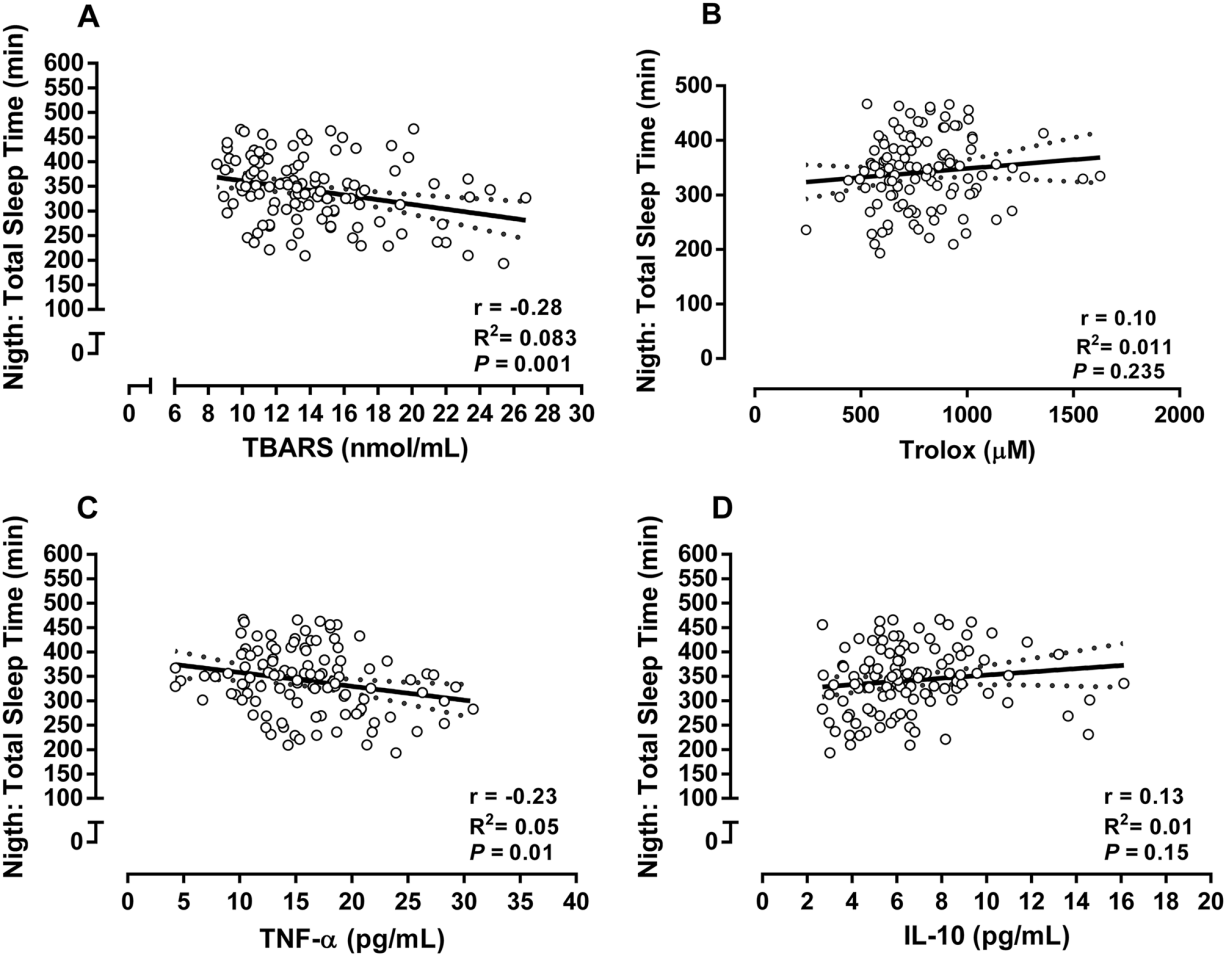
Pearson correlation coefficients to verify the association between redox and inflammatory profile with total sleep time. *TBARS* tiubarbituric acid reactive substances, *TNF-α* tumor necrosis factor alpha, *IL-10* interleukin 10.

Participants in the RTG increased NO_2_^−^ and ADMA decreased when compared to pre-training and CTL group, demonstrating a significant group × time interaction (*p* < 0.0001). The CTL group increased in ADMA across time (*p* < 0.0001). There was a significant, moderate, positive association of NO_2_^−^ with total sleep time (r = 0.50; *p* < 0.0001), and a significant, moderate, negative association between NO_2_^−^ and ADMA (r = − 0.66; *p* < 0.0001). See Fig. 4. There was a significant weak, negative association of TNF-α (r = − 0.23; *p* = 0.01) and TBARS (r = − 0.28; *p* = 0.001) with total sleep. See Fig. 5.

## Discussion

The current findings support the potential effect of a RT as a non-pharmacological strategy to improve total sleep time, sleep latency, nocturnal awakenings, and sleep efficiency in HD patients. Furthermore, RT enhanced redox and inflammatory profile in these patients, likely resulting from an increased bioavailability of NO and decreased ADMA concentration. Improving NO and ADMA may play an important role in the pathway to enhance the quality of sleep and health-associated factors in CKD patients.

Clinical professionals have seen the value of NO as a potent pharmacological outcome since it was discovered to be a endothelium-derived relaxing factor^19^; consequently, promoting cardiovascular benefits. NO is accepted as a regulator of blood pressure, platelet activation, and peripheral and central neurotransmission^20^. These physiological phenomenon occur because NO is an endogenous activator guanylate cyclase, leading to an increase of cyclic guanosine monophosphate concentrations, reducing cytosolic calcium concentration, and consequently, promoting vasodilatation^19–21^. Furthermore, NO plays a cytotoxic role in host defense against pathogens and tumor cells^20^. Building off what is already known about NO, the current investigation has reported that an increase of NO bioavailability seems to exert a protective effect against CKD and may be a potential pathway to improve to sleep quality in this population.

It is known that oxidative stress and inflammation are associated with a decrease in NO and increase in ADMA levels. Circulating ADMA is pointed as a possible mediator between CKD and sleep disorders as one study found HD patients presented lower sleep quality and higher ADMA levels^22^. However, others contend that the mechanisms associated with CKD and sleep disorder remain to be determined^22^. The current investigation has contributed data that suggest pro-inflammatory, oxidant factors, and ADMA are increased in the CTL group after the intervention, coinciding with a worsening of sleep quality. In contrast, the RT group increased the bioavailability of NO which can be explained by the decrease of inflammation, oxidative stress and ADMA, consequently, improving the quality of sleep in HD patients. We suggest that the redox balance, inflammation, and NO could be the cross-link between ADMA, CKD and sleep disorders. Indeed, low NO bioavailability seems to impair sleep quality in humans and animals, as it plays an important role in the regulatory mechanisms of the circadian clock, and modulation of encephalic centers of sleep^7^.

Both sleep quality and circadian rhythms are influenced mainly by aging. Older adults (included in the current investigation) experience fewer sleep hours and have more frequent awakenings^4^. These conditions directly impair health status and can lead to metabolic disturbances^9^. A key finding of the present study is the improvement of sleep patterns, which may minimize the impact of systemic, metabolic, and molecular disorders in HD patients. Buxton et al.^4^, observed that both young and older participants exposed to prolonged sleep restriction and circadian disruption experienced higher glucose levels and lower insulin responses to a standard meal. In this sense, the combined effect of elevated blood glucose and diminished insulin sensitivity increased the rate of progression of renal failure simply by affecting sleep^23^. The current RT intervention study demonstrates a possible non-pharmacological, alternative treatment strategy capable of improving sleep quality, which can stabilize imunometabolic profile in HD patients.

A comorbidity associated with CKD is obstructive sleep apnea^24^. Although it appears that NO increased in parallel to sleep quality with RT, promotion of NO can be contraindicated in population with history of obstructive sleep apnea^6,25^. Once NO is released in the nasal mucosa, patients often complain of congestion, increasing nasal resistance, decreased airway passage, and the frequency of airway collapse during sleep^25^. NO can play a dichotomous role in sleep disorders, either benefiting or harming, depending on the history of obstructive sleep apnea. In the current study, there was an increase in NO levels and also night total sleep time after 3 months of RT, which points to a close relationship between NO and sleep quality. Furthermore, must be highlighted that all subjects that participate of the present study was not diagnosticated with sleep apnea. To support these data, drugs that increased NO promoted improvements in sleep patterns in rodents; again, supporting a possible role of NO in mechanisms related to sleep control^8^. Unfortunately, the causality and physiological mechanisms are still to be elucidated in HD patients.

It has been reported that physical activity and increased energy expendidure improve sleep quality in HD patients^11^. Sakkas et al.^26^ demonstrated that intradialytic RT reduced body fat and improved glycemic homeostasis and sleep quality in this population. One possible explanation of this response, at least in part, would be the increase of a glucose uptake facilitator, NO, resultant of the exercise^27^.

While it appears that RT improves NO concentration, other factors may compete with this positive outcome. Reactive oxygen species induced by inflammation makes scavenger of NO, which become a more potential radical capable of promoting injuries in the cell structure such as DNA and proteins^28^. This increase in oxidative stress is accompanied by the rise of endogenous methylated arginine ADMA; which, chronically, can potentiate endothelial dysfunction and cardiovascular disease outcomes^29^. The current investigation demonstrated that RT has an essential role in improving redox balance, ADMA, and inflammation variables, all of which can increase the bioavailability of NO.

Despite great efforts, no study is void of limitations and opportunities for growth. The investigators recognize that the discomfort at the beginning of the RT program, despite a 2 week familiarization, likely lead to low effort in the RT group. However, all enrolled participants completed the intervention. The sample size may also limited the generalizability of the current study. Another limitation was that the sample of the present study is composed only by men, in this regard, further studies are important to verify the effect of RT on sleep disorders in HD women. Caution must be exercised before universally concluding that NO plays only a positive effect on sleep, as it appears to have a paradoxical role in worsening symptoms of obstructive sleep apnea.

In summary, we conclude that that RT may be practical in improving the clinical status of patients with CKD by improving their sleep quality, oxidative and inflammatory parameters. Further studies are required to elucidate the pathways of NO_2_^-^ in sleep disorders and other comorbidities.

## Methods

### Patients

**Figure 6:**
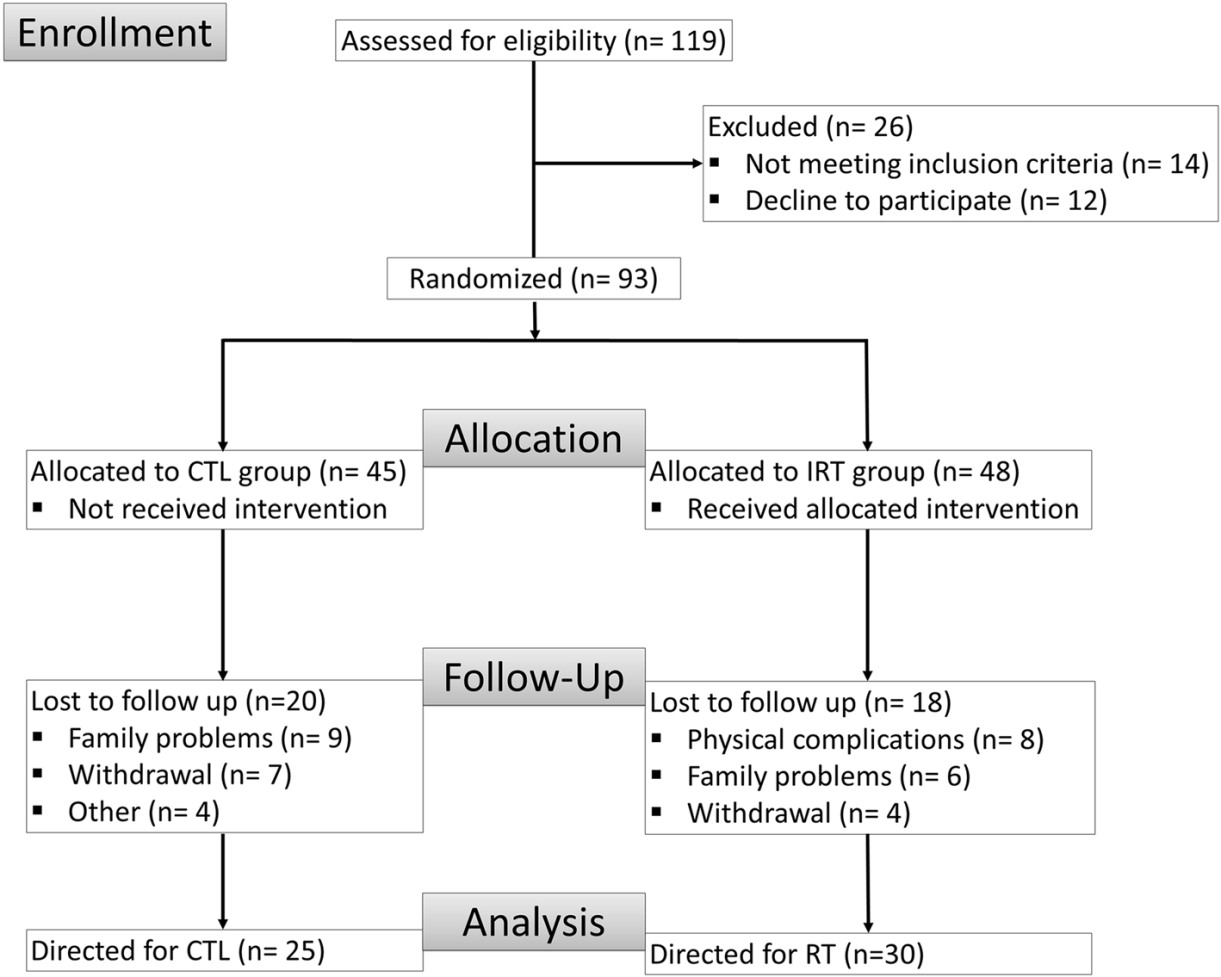
Flow chart of the present study. *CTL* control, *RT* resistance training.

Fifty-five men undergoing maintenance phase hemodialysis volunteered for this research investigation in January of 2019 until November of 2019. All experimental protocols were approved by Catholic University of Brasilia Ethics Committee, under the number: 23007319.0.0000.0029. This study was registered on the Brazilian clinical trials registration: URL: https://www.ensaiosclinicos.gov.br/rg/RBR-3gpg5w/, no RBR-3gpg5w (30/07/2019) and also registered in the World Health Organization international clinical trial resistry platform: URL: https://apps.who.int/trialsearch/utn.aspx, no U1111-1237-8231 (30/07/2019).Written informed consent is obtained from all the participants involved in the study. Inclusion criteria for participants were: (1) age ≥ 50 (once the prevalence of sleep disturbance is higher in aging plus CKD conditions); (2) on hemodialysis for at least 3 months; (3) dialysis at least three times per week; and (4) no significant medical complications in the last 3 months, with the exception of vascular access correction. Exclusion criteria were: (1) recent acute myocardial infarction within 3 months or unstable angina; (2) systemic lupus erythematosus; (3) congenital kidney malformation or some autoimmune disease that affects the kidneys; (4) osteoarticular complications that could compromise physical exercise; (5) decompensated heart failure that could limite participation in training; (6) severe decompensated diabetes; and (7) severe neuropathy, retinopathy, or diabetic nephropathy. Only patients who read, agreed, and signed the written informed consent participated in this study. The participants were then randomized into two groups control group (CTL, n = 25) and resistance training group (RTG, n = 30). The randomization was a simple randomization perfomed by just one resercher using a random number generator. All patients underwent nutritional instruction by the clinical nutritionist. Losses and exclusion are described in Fig. 6. The authors declare that all procedures were carried out in accordance with the guidelines of the American College in Sports Medicine in exercise testing and prescription^30^. Moreover, the present study is in accordance with the guidelines and regulation for exercise prescription for end-stage renal disease^31^.

### Resistance training intervention

Patients in the RTG participated in a periodized RT program structured in 50 min sessions, three sessions per week for 12 weeks while the patients were receiving dialysis (intradialytic exercise). RT repetitions balanced concentric and eccentric lifting phases (each phase lasted 2 s), verified and supervised by a strength and conditioning specialist. RTG participants completed 2 weeks of familiarization designed to minimize the discomfort during training sessions, which took place within the first 2 h of dialysis.

The RTG sessions consisted of the following eleven exercises: chest press with Thera-band and rowing with Thera-band (dynamometer E-LASTIC); dumbbell biceps curl; unilateral overhead triceps extension; unilateral shoulder abduction, and shoulder press with dumbbells (shoulder development); bilateral knee extension with weights attached to the ankles; weight hip thrust; hamstrings curl with Thera-band (dynamometer E-LASTIC); hip adduction with Thera-band (dynamometer E-LASTIC); hip abduction with Thera-band (dynamometer E-LASTIC), and seated plantar flexion with fixed weights ranging from 2 to 15 kg. Upper body exercises were performed with the limb without the arteriovenous fistula. In all exercises, a rating or perceived exertion of 6 and 8 (somewhat hard to hard) according to OMNI-RES scale was established for training overload, periodization, and progression^32^. RTG was programmed for three sets of 8–12 repetitions with 2 min of rest between sets.

### Sleep quality assessment

Accelerometers (ACTTRUST model; Condor Instruments, Ltda, São Paulo—Brazil) were worn on the non-dominant wrist to assess sleep quality. Data detection and recording were performed using the wake threshold selection method. This strategy, unlike the zero-crossing process, cumulatively computes the duration and quantity of times movement exceeded the manufacturer’s average sleep/awake threshold^33^. There appears to be approximately 90% agreement between polysomnography (gold standard) and the implemented algorithm^34^. The criterion used to calculate the sleep interval was ten minutes of immobility from the beginning and end of sleep. The subjects were instructed to wear the accelerometer continuously across nine consecutive days. The first and last days were discarded to reduce risk of bias^34^, leaving 7 days and seven nights for the final evaluation. The following variables were gathered from the accelerometer: total sleep at night time (minutes), sleep latency (amount of time it takes to transition from waking to full sleep), nocturnal awakenings, and sleep efficiency (number of hours spent in bed and number of hours actually slept). Participants were debriefed by a group of psychologists who explained the importance of the results after completing the trial.

### Biochemical analysis

Venous blood samples were collected at baseline and after 12 weeks of training for interleukin 10 (IL-10), tumor necrosis factor-alpha (TNF-α), nitric oxide (NO), thiobarbituric acid reactive substances (TBARS), total antioxidant capacity (Trolox equivalent), and asymmetric dimethylarginine (ADMA). The samples for biochemical analysis were obtained in the morning (average time was 8:00 a.m.) centrifuged at 1,500×*g* for 15 min; after processing, the specimens were aliquoted into cryovials and stored at − 80 °C. The systemic levels of TNF-α and L-10 were measured in triplicate by enzyme-linked immunosorbent assay (ELISA) kits from R&D Systems (Minneapolis, MN, USA) according to the manufacturer’s instructions. The detectable limit for TNF-α and IL-10 were ten pg/mL and 0.2 pg/mL, respectively. The overall intra- and inter-assays CVs for inflammatory markers were less than 10%. TBARS, total antioxidant capacity (Trolox equivalent), and NO bioavailability (assessed by NO_2_^−^ levels) were analyzed as previously described by Sousa et al.^35^ ADMA concentration was determined by ELISA, the intra- and inter-assay coefficients of variation (CV) were ≤ 8% and ≤ 12%, respectively (Human ADMA ELISA Kit, MyBioSource, San Diego, USA).

### Statistical analysis

The primary aim of the present study was to evaluate the effect of RT on sleep quality. A secondary purpose was to investigate the effect of RT on NO, metabolism markers, and inflammatory and redox profiles. An a priori power analysis was calculated with G*power (version 3.1.9.4), an alpha of 5%, presenting a power of 0.950. The normality and homogeneity of data were tested by the Shapiro–Wilk and Levene tests, respectively. Data were expressed as means and standard deviations. Chi-square test was used to analyze the baseline characteristics of individuals. A one-way repeated measures (RM) ANOVA followed the Tukey’s post-hoc test were utilizated to evaluate total work differences between weeks. Groups were compared at pre- and post-training by a two-way RM ANOVA (within and between factors) with Tukey’s post-hoc tests as needed. Pearson correlation coefficients were calculated for both groups (CTL and RT) in both periods (pre and post) together to evaluate the relationship of NO to ADMA and NO, redox balance and inflammatory profile to total sleep time. Statistical significance was accepted at *p* < 0.05. Statistical analyses were performed using the GraphPadPrism6.0 (San Diego, USA).

## Data Availability

Data are available upon request.
